# Differentiation in MALDI-TOF MS and FTIR spectra between two closely related species *Acidovorax oryzae* and *Acidovorax citrulli*

**DOI:** 10.1186/1471-2180-12-182

**Published:** 2012-08-18

**Authors:** Yanli Wang, Qing Zhou, Bin Li, Baoping Liu, Guoxing Wu, Muhammad Ibrahim, Guanlin Xie, Hongye Li, Guochang Sun

**Affiliations:** 1 State Key Laboratory of Rice Biology, Institute of Biotechnology, Zhejiang University, Hangzhou, 310029, China; 2 State Key Laboratory Breeding Base for Zhejiang Sustainable Pest and Disease Control, Institute of Plant Protection and Microbiology, Zhejiang Academy of Agricultural Sciences, Hangzhou, 310021, China; 3 College of Plant Protection, Yunnan Agricultural University, Kunming, 650201, China

## Abstract

**Background:**

Two important plant pathogenic bacteria *Acidovorax oryzae* and *Acidovorax citrulli* are closely related and often not easy to be differentiated from each other, which often resulted in a false identification between them based on traditional methods such as carbon source utilization profile, fatty acid methyl esters, and ELISA detection tests. MALDI-TOF MS and Fourier transform infrared (FTIR) spectra have recently been successfully applied in bacterial identification and classification, which provide an alternate method for differentiating the two species.

**Results:**

Characterization and comparison of the 10 *A. oryzae* strains and 10 *A. citrulli* strains were performed based on traditional bacteriological methods, MALDI-TOF MS, and FTIR spectroscopy. Our results showed that the identity of the two closely related plant pathogenic bacteria *A. oryzae* and *A. citrulli* was able to be confirmed by both pathogenicity tests and species-specific PCR, but the two species were difficult to be differentiated based on Biolog and FAME profile as well as 16 S rRNA sequence analysis. However, there were significant differences in MALDI-TOF MS and FTIR spectra between the two species of *Acidovorax*. MALDI-TOF MS revealed that 22 and 18 peaks were specific to *A. oryzae* and *A. citrulli*, respectively, while FTIR spectra of the two species of *Acidovorax* have the specific peaks at 1738, 1311, 1128, 1078, 989 cm^-1^ and at 1337, 968, 933, 916, 786 cm^-1^, respectively.

**Conclusions:**

This study indicated that MALDI-TOF MS and FTIR spectra may give a new strategy for rapid bacterial identification and differentiation of the two closely related species of *Acidovorax*.

## Background

Many researches have focused on the specific detection of the two important plant pathogenic bacteria *Acidovorax oryzae* (formerly *Acidovorax avenae* subsp. *avenae*) and *Acidovorax citrulli* (formerly *Acidovorax avenae* subsp. *citrulli*) [1,2]. However, the two species are closely related and often not easy to be differentiated from each other [3,4], which often resulted in a false identification between them based on traditional methods such as carbon source utilization profile, fatty acid methyl esters, PCR and ELISA detection tests [1,5]. Therefore, it is necessary to develop an alternate method for differentiating the two species.

Recently, MALDI-TOF MS and Fourier transform infrared (FTIR) spectra have been successfully applied in bacterial identification and classification [6-11]. MALDI-TOF MS allows bacterial identification at the species level by measuring molecular masses of proteins and other bacterial components obtained from whole bacterial extracts, while FTIR spectroscopy allows the analysis of small quantities of biomass, simultaneous characterization of different functional groups such as lipids, proteins, nucleic acids and polysaccharides in biological molecules and complex structures and without disturbing the systems, and requires no consumables or reagents [6,12,13]. However, little information was obtained about the applications of MALDI-TOF MS and FTIR spectra in plant pathogenic bacteria.

The objective of this study was to examine and compare the MALDI-TOF MS and FTIR spectra of bacteria from the two species of *Acidovorax*.

## Methods

### Bacterial strains

The 10 virulent strains of *A. oryzae* used in this study were isolated from diseased rice seed and seedling, while the 10 virulent strains of *A. citrulli* were isolated from diseased watermelon and melon (Table 1). The identities of bacterial strains were determined and confirmed based on the biochemical and physiological characteristics as described by Krieg and Holt [14] and Schaad et al. [15], whole-cell fatty acid and Biolog metabolic assays as described by Li et al. [3,16,17], species-specific PCR[1,15,18] and 16 S ribosomal RNA gene sequence analysis [3,16,17]. The representative *A. oryzae* strain R1001 (Collection no: ACCC05733) and *A. citrulli* strain Ab1 (Collection no: ACCC05732) were deposited in Agricultural Culture Collection of China (ACCC).

**Table 1:** **Strains of *Acidovorax oryzae* (Ao) and *Acidovorax citrulli* (Ac) used in this study**

**Ao strains**	**Sources**	**Ac strains**	**Sources**
R1001	Rice seedling, this lab	A1	Watermelon leaf, CAAS, China
R1002	Rice seedling, this lab	Aacf	Watermelon leaf, FAFFU, China
R1003	Rice seedling, this lab	Ab1	Watermelon leaf, this lab
R1004	Rice seedling, this lab	Njf4	Watermelon leaf, NAU, China
CB97012	Rice seeds, this lab	Ps96	Watermelon leaf, CAAS, China
CB97058	Rice seeds, this lab	Ab3	Melon leaf, this lab
CB97063	Rice seeds, this lab	Tw20	Melon leaf, CAAS, China
CB97181	Rice seeds, this lab	Ab5	Melon leaf, this lab
CB97095	Rice seeds, this lab	Ab8	Melon leaf, this lab
CB97128	Rice seeds, this lab	Ab9	Melon leaf, this lab

CAAS: Chinese Academy of Agricultural Sciences; FAFFU: Fujian Agricultural and Forestry University; NAU: Nanjing Agricultural University.

### MALDI-TOF MS

#### Sample preparation

One loop of bacterial cells grown on Luria-Bertani at 30°C for 48 h was suspended in 300 μl of Millipore water followed by adding 900 μl of absolute ethanol. Cell pellets were obtained by a centrifugation at 12000 rpm for 2 min and suspended in 50 μl of formic acid (70% v/v) followed by carefully adding 50 μl of acetonitrile. One microliter of supernatant after a centrifugation at 12000 rpm for 2 min was spotted on a steel target plate (Bruker Daltonic, Billerica, Massachusetts) and air dried at room temperature. Afterwards, 1 μl of matrix solution (saturated solution of α-cyanohydroxycinnaminic acid in 50% aqueous acetonitrile containing 2.5% trifluoroacetic acid) was quickly added onto the surface of each sample spot. Samples were prepared in duplicate.

### MALDI-TOF MS analysis

Mass spectrometric measurements were preformed with an AUTOFLEX Analyzer (Bruker Daltonics) as described in previous studies using the linear positive ion extraction [10,11,19]. The method of identification included the *m/z* from 2 to 12 kDa. *Escherichia coli* DH5α was used as an external protein calibration mixture followed by the Bruker Test Standard [20]. Raw mass spectrum smooth, baseline correction and peak detection were performed using the corresponding programs installed in the MS system. Resulting mass fingerprints were exported to FLEX ANALYSIS (Bruker Daltonics) and analyzed. Spectral data were investigated for the presence of biomarkers characteristic for each of the two *Acidovorax* species. After visual inspection and comparison, the most intensive and predominantly present protein peaks were selected and screened in representatives of each species.

### FTIR spectroscopy

#### Sample preparation

Bacterial cells were collected from overnight Luria-Bertani broth culture grown at 28°C by centrifugation at 10,000 rpm for 10 min. After removing the supernatants, the bacterial pellets were washed twice with double distilled water. After second wash in double distilled water, bacterial samples were stored at −70°C until lyophilisation. The samples for FTIR analysis were first grounded into fine particles using mortar and pestle. The 1 mg of each sample was then mixed with 100 mg potassium bromide (KBr) which extensively dried in microfuge tubes using a lyophiliser. These mixtures have been dried for an additional 2 h in the same microfuge tubes. The KBr based pellets were then compressed into a thin disk by establishing pressure of 100 kg/cm^2^ (1200 psi) for about 8 min.

### FTIR spectroscopy and data analysis

The FTIR spectroscopy data were analysed as previously described by Garip et al. [21] with a small modification. Pellets were scanned at 4 cm^-1^ resolution with 100 scans in the spectral range of 4000–500 cm^-1^ at room temperature. The sample compartment in the FTIR spectrometer was continuously purged with dry air to prevent water vapour. Analysis of the spectral data was performed by using Grams 32 (Galactic Industries, Salem, NH, USA) software. The spectral range of 4000–500 cm^-1^ was analyzed. The band positions were measured according to the center of weight. The averages of the spectra belonging to the same experimental groups, baseline correction, normalisation and the band areas were obtained by using the same software program. The average spectra and normalisation process were applied only for visual representation of the differences, however for the determination of the spectral parameters and calculation of mean values and statistical analysis each baseline corrected original spectrum was taken into consideration.

### Statistics

The software STATGRAPHICS Plus, version 4.0 (Copyright Manugistics Inc., Rockville, Md., USA) was used to perform the statistical analysis. Levels of significance (*p* < 0.05) of main treatments and their interactions were calculated by analysis of variance after testing for normality and variance homogeneity.

## Results and discussion

### Bacterial identity

Results from this study indicated the rice strains should be identified as *A. oryzae* with Biolog similarity of 0.72 to 0.73, FAME similarity of 0.73 to 0.74, 16 S rRNA sequence similarity of 99% and confirmed by both pathogenicity tests and species-specific PCR, while the watermelon and melon strains should be identified as *A. citrulli* with Biolog similarity of 0.70 to 0.73, FAME similarity of 0.73 to 0.74, 16 S rRNA sequence similarity of 99%, and confirmed by both pathogenicity tests and species-specific PCR in the newly proposed classification of subspecies of *A. avenae*. However, in general, the two species of *Acidovorax* were high similar, and difficult to be differentiated based on Biolog and FAME profile as well as 16 S rRNA sequence analysis*.*

### MALDI-TOF MS

MALDI-TOF MS characterization of bacteria is based on differences in mass to charge ratio (m/z) fingerprints of whole cell proteins, mainly representing ribosomal proteins which are most abundantly expressed under all growth conditions [22]. In this type of mass spectrometry, samples were prepared by embedding analyte molecules in a crystal matrix of small acidic molecules. A brief laser pulse irradiates the sample and the matrix absorbs the laser energy resulting in ablation of a small volume of matrix and desorption of the embedded analyte molecules which are ionized. Subsequently, predominantly single charged analyte ions can be detected and analyzed [23].

**Figure 1:**
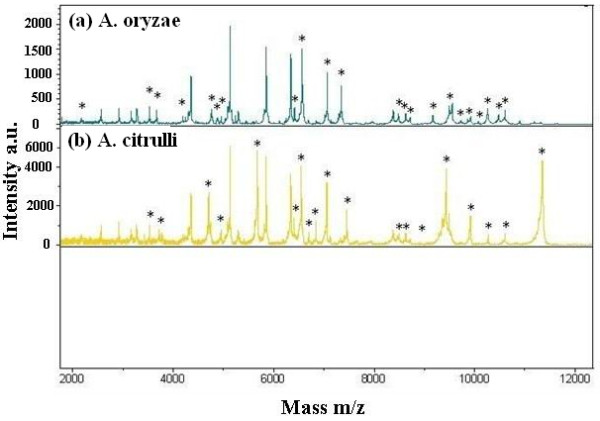
**MALDI-TOF MS protein mass fingerprints of *Acidovorax oryzae* and *Acidovorax citrulli.* ** Similar and different marker masses for the identification of *A. oryzae* and *A. citrulli* are listed in Table 2. Intensity of ions is shown on the y axis and the mass (in Daltons) of the ions is shown on the x axis. The m/z values represent mass-to charge ratios. *: Unique peaks positions for each of species.

Figure 1 presents a typical MALDI-TOF MS spectrum for the two species, which contain a contiguous sequence of about high-intensity ion peaks between 2000 and 12,000 Da. The obtained spectral profiles were further screened for the presence of recurring peaks or biomarker ions specific for both the species. Fifty selected m/z values were summarized in Table 2, while ten m/z values were detected in both species, making them characteristic for the genus *Acidovorax*. In addition, MALDI-TOF MS revealed that 22 and 18 peaks were specific to *A. oryzae* and *A. citrulli*, respectively (Table 2, Figure 1). These unique peaks for each species offer a strong proof in differentiating the two species*.* This result is consistent with the review of Moore et al. [24], which found that MALDI-TOF MS is a valuable and reliable tool for microbial identification in a number of studies.

**Table 2:** **Characteristic MALDI-TOF masses (in Daltons) selected as possible biomarkers for identification of *Acidovorax oryzae* (Ao) and *Acidovorax citrulli* (Ac)**

**Ao**	**Ac**	**Ao**	**Ac**
2178			6703
**2568**	**2565**		6845
**2932**	**2930**		7055
**3169**	**3168**	7067	
**3281**	**3285**	7349	
	3524		7461
3533		**8387**	**8381**
3675		8486	
	3729		8494
4184			8636
**4353**	**4351**	8642	
	4716	8709	
4777		9181	
4885		9545	
	4956		9503
4965		9746	
**5135**	**5133**		9919
**5304**	**5305**	9935	
	5674	10097	
**5863**	**5861**	10260	
**6339**	**6337**		10271
	6413	10503	
6420			10608
	6550	10609	
6568			11349

Masses observed in both species are marked in bold while species unique mass values marked in Figure 1. Assigned proteins calculated using RMIDb.

### FTIR spectroscopy

**Figure 2:**
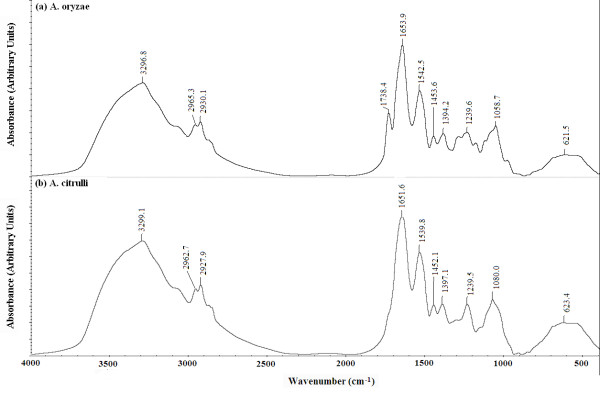
**The average FTIR spectra in the 4000–500 cm**^**-1**^**region for both *Acidovorax oryzae* (n = 10) and *Acidovorax citrulli* (n = 10).**

In agreement with the result of bacteriological characterization, the 10 strains of *A. oryzae* had a very similar FTIR spectrum while the 10 strains of *A. citrulli* had a very similar FTIR spectrum regardless of bacterial origin (data not shown), indicating the stability and reliability of the FTIR spectroscopic system. In addition, characterization and differentiation of the two species were performed based on the average FTIR spectrum of the 10 *A. oryzae* strains and the 10 *A. citrulli* strains, respectively (Figure 2). In general, there were significant differences in both the frequency and the intensity values of the 10 main functional groups between the two species except the frequency of PO_2_^-^ asymmetric stretching (Table 3), which indicated that the method of FTIR spectrum maybe have a higher level of differentiation between the two species compared to the biochemical and physiological characteristics tested in this study.

**Table 3:** **The band frequencies and absorption intensity of various functional groups in the *Acidovorax oryzae* (Ao) and *Acidovorax citrulli* (Ac) strains**

**Functional groups**	**Frequency (cm**^**-1**^**)**			**Intensity**		
	**Ao (n = 10)**	**Ac (n = 10)**	** *P* -value**	**Ao (n = 10)**	**Ac (n = 10)**	** *P* -value**
CH_3_ asymmetric stretching	2965.25 ± 0.35	2962.68 ± 0.14	^***^	1.14 ± 0.02	1.19 ± 0.02	^*^
CH_2_ asymmetric stretching	2930.14 ± 0.26	2927.85 ± 0.23	^***^	1.23 ± 0.02	1.25 ± 0.01	^*^
CH_3_ symmetric stretching	2873.22 ± 0.47	2875.97 ± 0.36	^***^	0.83 ± 0.01	0.89 ± 0.02	^*^
CH_2_ symmetric stretching	2853.15 ± 0.36	2855.22 ± 0.56	^***^	0.74 ± 0.05	0.86 ± 0.07	^**^
Amide I	1653.85 ± 0.21	1651.61 ± 0.14	^***^	2.97 ± 0.15	1.84 ± 0.25	^***^
Amide II	1542.53 ± 0.33	1539.82 ± 0.11	^***^	1.98 ± 0.25	1.57 ± 0.36	^**^
CH_2_ bending	1453.61 ± 0.43	1452.14 ± 0.14	^**^	0.90 ± 0.03	0.96 ± 0.02	^*^
COO^-^ symmetric stretch	1394.20 ± 0.36	1397.09 ± 0.25	^***^	0.98 ± 0.02	0.92 ± 0.05	^*^
PO_2_^-^ asymmetric stretching	1239.61 ± 0.12	1239.48 ± 0.19	0.12	1.01 ± 0.02	0.91 ± 0.02	^*^
PO_2_^-^ symmetric stretching	1058.65 ± 1.78	1080.02 ± 0.56	^***^	1.14 ± 0.19	0.89 ± 0.08	^***^

Data are the mean of the 10 strains. ^*^: p < 0.05, ^**^: p < 0.01, ^***^: p < 0.001.

The average spectra in the 4000–500 cm^-1^ region indicated that the *A. oryzae* strains have a higher frequency of the CH_3_ asymmetric stretching vibration at 2959 cm^-1^, the CH_2_ asymmetric stretching vibration at 2927 cm^-1^, the Amide I band at 1657 cm^-1^, Amide II band at 1541 cm^-1^, and the CH_2_ bending band at 1452 cm^-1^ compared to the *A. citrulli* strains, while the *A. citrulli* strains have a higher frequency of the CH_3_ symmetric stretching vibration at 2876 cm^-1^, the CH_2_ symmetric stretching vibration at 2857 cm^-1^, the COO^-^ symmetric stretch band at 1391 cm^-1^ and the PO_2_^-^ symmetric stretching; phospholipids C-O stretch band at 1080 cm^-1^ compared to the *A. oryzae* strains (Figure 2; Table 3; Additional file 1). In addition, the *A. oryzae* strains have a higher intensity of the absorption in the Amide I band at 1657 cm^-1^, Amide II band at 1541 cm^-1^, the COO^-^ symmetric stretch band at 1391 cm^-1^, the PO_2_^-^ asymmetric stretching band at 1236 cm^-1^, the PO_2_^-^ symmetric stretching; phospholipids C-O stretch band at 1080 cm^-1^ compared to the *A. citrulli* strains, while the *A. citrulli* strains have a higher intensity of the absorption in the CH_3_ asymmetric stretching vibration at 2959 cm^-1^, the CH_2_ asymmetric stretching vibration at 2927 cm^-1^, the CH_3_ symmetric stretching vibration at 2876 cm^-1^, the CH_2_ symmetric stretching vibration at 2857 cm^-1^, the CH_2_ bending band at 1452 cm^-1^ compared to the *A. oryzae* strains. However, there was not significant difference in the frequency value of the PO_2_^-^ asymmetric stretching band at 1236 cm^-1^ between the two species (Figure 2; Table 3; Additional file 1)*.*

The average spectra in the 2800–1800 cm^-1^ region were not detailed compared between the two species for no obvious peaks were found in the region (Figure 2; Table 3). Interestingly, this result indicated that five distinctive peaks around at 1738, 1311, 1128, 1078 and 989 cm^-1^ was observed in the *A. oryzae* strains, but not in the *A. citrulli* strains, while five distinctive peaks centered at 1337, 968, 933, 916 and 786 cm^-1^ was only observed in the *A. citrulli* strains, but not in the *A. oryzae* strains (Figure 2; Table 3; Additional file 1). These characteristic peaks are specific to either the *A. citrulli* strains or the *A. oryzae* strains. Therefore, it could be suggested that these characteristic peaks may be able to be used for the discrimination of the two species of *Acidovorax*.

Previous related reports have revealed that the prominent peak centered at 2959 cm^-1^ is mainly due to lipids, the prominent peak centered at 2927 cm^-1^ is mainly due to lipids and with little contribution from proteins, carbohydrates and nucleic acids, the prominent peak centered at 2876 cm^-1^ is mainly due to proteins, the prominent peak centered at 2857 cm^-1^ is mainly due to lipids, the band centered at 1739 cm^-1^ is mainly assigned to the C = O ester stretching vibration of triglycerides, the bands centered at 1657 cm^-1^ is mainly assigned to the stretching C = O (amide I) vibrational modes of the polypeptide and protein backbone, the band centered at 1541 cm^-1^ is mainly assigned to the bending N-H and stretching C-N (amide II), the band at 1452 cm^-1^ is mainly assigned to the CH_2_ bending mode of lipids [6-9,12,13,25-29], the band at around 1337 cm^-1^ was due to acetic acid which was produced by an acetate oxidation [30], the bands at 968, 933 and 916 cm^-1^ were assigned to the vibration of C-O-C ring deoxyribose, the lipid C = O stretching vibration band at 1738 cm^-1^ has been suggested as indicative of an increased concentration and difference in packing of the ester groups in bacteria [31]. Furthermore, the band at 1311 cm^-1^ was due to the stretching mode of C–O of carboxylic acids which suggested an exopolymer formation in bacteria [32], while these bands at 1128, 1078 and 989 cm^-1^ were due to DNA and RNA backbones, glycogen, and nucleic acids, respectively [6,21]. Therefore, the difference of FTIR spectra between the two species may be due mainly to the imparity of the macromolecular composition and concentration.

This study revealed that the protein-to-lipid ratio was significantly higher for the *A. oryzae* strains than for the *A. citrulli* strains in this study (Figure 2; Table 4), which was calculated by taking the ratio of the area of the CH_3_ symmetric stretching band at 2876 cm^-1^ to the area of the CH_3_ asymmetric stretching band at 2959 cm^-1^[6,21]. In agreement with the result of the protein-to-lipid ratio, the ratio of DNA-to-protein was higher for the *A. citrulli* strains than for the *A. oryzae* strains (Figure 2; Table 4), which was calculated by taking the ratio of the area of PO_2_^-^ symmetric stretching band at 1080 cm^-1^ to the area of the band at 1541 cm^-1^[6,21].

**Table 4:** **The band area values of various functional groups and protein/lipid ratio values in *Acidovorax oryzae* (Ao) and *Acidovorax citrulli* (Ac) strains**

**Functional groups**	**Ao (n = 10)**	**Ac (n = 10)**	**P-value**
Band area value			
CH_3_ asymmetric stretching	0.152 ± 0.002	0.183 ± 0.010	^*^
CH_3_ symmetric stretching	0.053 ± 0.004	0.036 ± 0.002	^*^
Amide I	3.603 ± 0.021	1.668 ± 0.036	^***^
Amide II	1.931 ± 0.012	1.150 ± 0.011	^**^
PO_2_^-^ asymmetric stretching	0.379 ± 0.062	0.801 ± 0.008	^**^
PO_2_^-^ symmetric stretching	1.061 ± 0.051	1.182 ± 0.036	^**^
Protein/lipids ratio			
CH_3_ symmetric/CH_3_ asymmetric	0.349 ± 0.044	0.196 ± 0.015	^***^
DNA/Protein ratio			
PO_2_^-^ asymmetric/Amide II	0.196 ± 0.006	0.697 ± 0.007	^***^

Data are the mean of the 10 strains. ^*^: p < 0.05, ^**^: p < 0.01, ^***^: p < 0.001.

The ratio of protein-to-lipid in the membranes is an important factor affecting the membrane structure and dynamics [33]. Interestingly, the frequency of Amide I and Amide II has been regarded as indicative of conformation and structure of cellular proteins [31,34], while the absorption intensity of Amide I and Amide II has been regarded as indicative of protein content in bacterial cells [6,21]. However, in this study, the *A. oryzae* strains not only have a higher value in the frequency and the absorption intensity of both Amide I and Amide II, but also in the triglyceride content that is indicative of the lipids compared to the *A. citrulli* strains. Therefore, the major contribution to the higher protein-to-lipid ratio in the *A. oryzae* strains comes from the significant increase of the area of both Amide I and Amide II.

## Conclusions

In summary, our results indicated that there were significant differences in MALDI-TOF MS and FTIR spectra between the two species. In particular, several specific characteristic peaks were determined for each of the two species. Compared to the traditional time-consuming method, MALDI-TOF MS and FTIR spectroscopy is easy to implement and is an emergent physico-chemical technique in bacterial research. Therefore, result from this study may give a new strategy for the rapid bacterial identification and differentiation of the two species of *Acidovorax*.

## Competing interests

None declared.

## Authors’ contributions

Wang YL and Li B designed the experiments and wrote the paper. Liu BP, Zhou Q, Wu GX and Ibrahim M performed the experiments. Xie GL, Li HY and Sun GC coordinated the project. All authors have read and approved the manuscript.

## Supplementary Material

Additional file 1 **The average FTIR spectra in the 4000–2800 cm**^**-1**^**(a); 1800–1400 cm**^**-1**^**(b); 1400–1000 cm**^**-1**^**(c); 1000–500 cm**^**-1**^**(d) region for both *Acidovorax oryzae* (n = 10) and *Acidovorax citrulli*  (n = 10).** Click here for file
