# Supplementary figures and images for: Differentiation in MALDI-TOF MS and FTIR spectra between two closely related species Acidovorax oryzae and Acidovorax citrulli
Source: BMC Microbiol. 2012 Aug 18;12:182. doi: 10.1186/1471-2180-12-182 (PMC3438124; doi:10.1186/1471-2180-12-182)

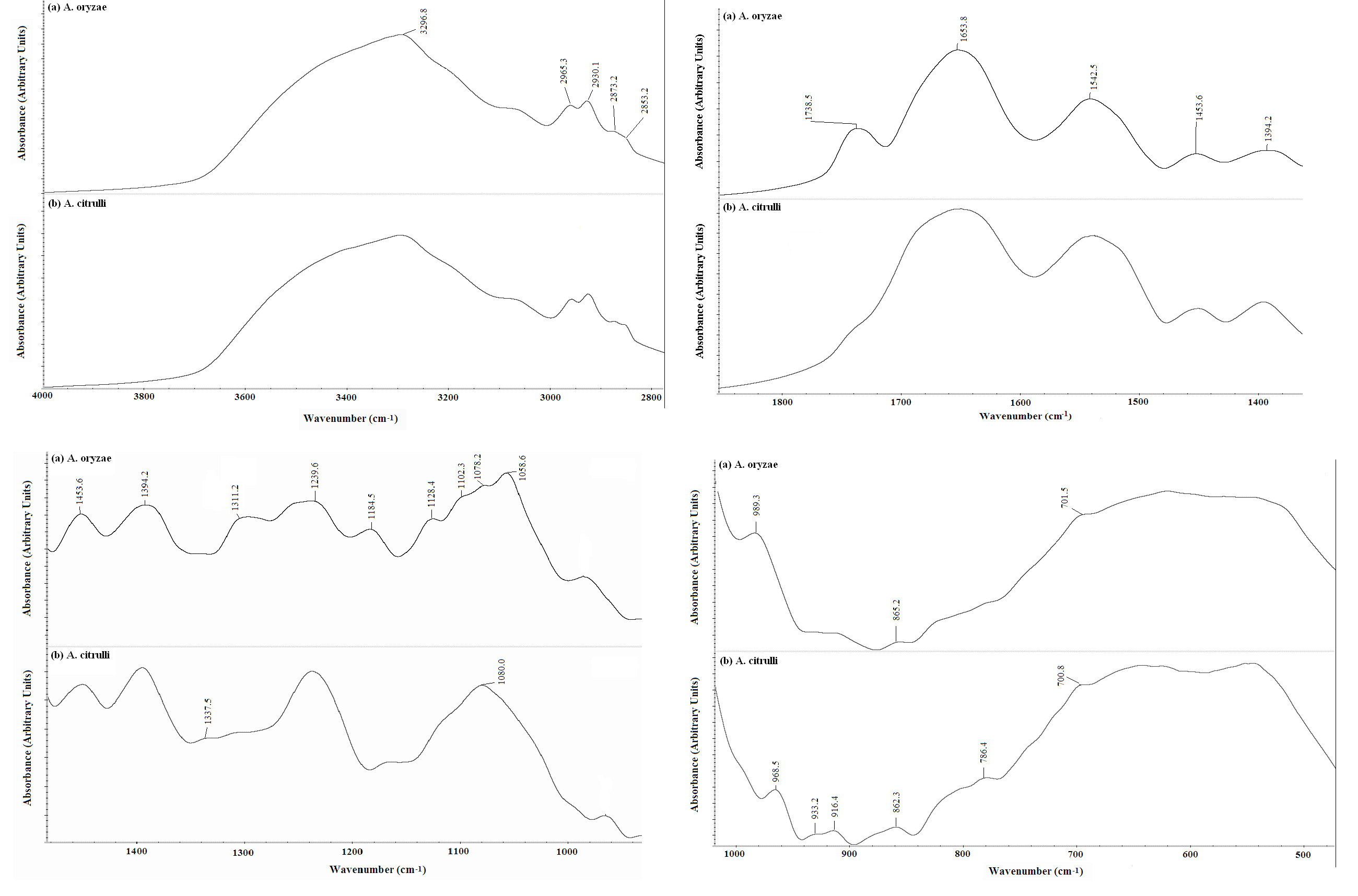

Supplement: Additional file 1 — The average FTIR spectra in the 4000–2800 cm-1(a); 1800–1400 cm-1(b); 1400–1000 cm-1(c); 1000–500 cm-1(d) region for both Acidovorax oryzae (n = 10) and Acidovorax citrulli (n = 10). [file 1471-2180-12-182-S1.tiff]
